# The Mental Health Impact of Computer and Internet Training on a Multi-ethnic Sample of Community-Dwelling Older Adults: Results of a Pilot Randomised Controlled Trial

**Published:** 2013-09

**Authors:** Luciana Laganá, James J. García

**Affiliations:** 1 Department of Clinical Psychology, California State University Northridge, Northridge, California, USA;; 2 Department of Psychology, University of North Texas, Denton, Texas, USA

**Keywords:** Older adults, Ethnic diversity, Depression, Self-esteem, Computer attitudes, Computer self-efficacy, Computer technology training

## Abstract

**Introduction:**

We preliminarily explored the effects of computer and internet training in older age and attempted to address the diversity gap in the ethnogeriatric literature, given that, in our study’s sample, only one-third of the participants self-identified as White. The aim of this investigation was to compare two groups - the control and the experimental conditions - regarding theme 1) computer attitudes and related self-efficacy, and theme 2) self-esteem and depressive symptomatology.

**Methods:**

Sixty non-institutionalized residents of Los Angeles County (mean age ± SD: 69.12 ± 10.37 years; age range: 51-92) were randomly assigned to either the experimental group (n=30) or the waitlist/control group (n=30). The experimental group was involved in 6 weeks of one-on-one computer and internet training for one 2-hour session per week. The same training was administered to the control participants after their post-test. Outcome measures included the four variables, organized into the two aforementioned themes.

**Results:**

There were no significant between-group differences in either post-test computer attitudes or self-esteem. However, findings revealed that the experimental group reported greater computer self-efficacy, compared to the waitlist/control group, at post-test/follow-up [*F*(1,56)=28.89, *p*=0.001, *η_2_*=0.01]. Additionally, at the end of the computer and internet training, there was a substantial and statistically significant decrease in depression scores among those in the experimental group when compared to the waitlist/control group [*F*(1,55)=9.06, *p*<0.004, *η_2_*=0.02].

**Conclusions:**

There were significant improvements in favour of the experimental group in computer self-efficacy and, of noteworthy clinical relevance, in depression, as evidenced by a decreased percentage of significantly depressed experimental subjects from 36.7% at baseline to 16.7% at the end of our intervention.

## INTRODUCTION

In the present study, we were interested in testing the effects of a computer technology training intervention on older adults’ computer technology comfort and well–being. It is particularly important to investigate ways to decrease depressive symptomatology regardless of age, as one of the most common psychological disorders for all Americans is depression (1). Its prevalence within senior populations, the target of the present study, is high, with about 5 million older adults in the U.S.A. experiencing some form of persistent depressive symptomatology. Between 5% and 10% of older adults who visit their primary care physicians are depressed (2, 3). Moreover, research in this area should target non-White populations, as individuals of racial and ethnic minority backgrounds over the age of 65 represent a rapidly growing segment of the U.S. population, currently totaling over 13% (4). Specific to the location of our study, in a 2008 investigation surveying 16,500 older adults residing in Los Angeles County (5), compared to White seniors, older adults from racial/ethnic groups reported higher rates of a variety of unmet needs such as greater health needs, employment needs, social isolation concerns, as well as housing, transportation, and caregiving needs. Hispanic/Latino residents, who were among those reporting the most unmet needs, stated that daily activities, in particular, were a problematic issue: a phenomenon which could certainly contribute to depressive symptomatology in this population.

In addition to the troubling prevalence of depressive symptomatology in older age, self-esteem can decrease in older age due to social role losses, reduction of physical beauty, decreased health, and related deficits. The inclusion of self-esteem in studies on interventions for geriatric depression is methodologically appropriate and conceptually pertinent, given that there is a strong inverse association between self-esteem and depression in many age groups (6, 7). Among the few geriatric studies on this issue, one study (8) has shown a significant inverse relationship between these two variables among older adults with orthopedic disabilities. A more dated study on correlates of self-esteem (9) revealed that seniors with low self-esteem had significantly more depression than those with high self-esteem. The authors of the aforementioned study, based on their findings, highlighted the need to develop interventions that promote enhanced self-image in older age. Another reason for targeting self-esteem in geriatric interventions is that, as it has been suggested in the literature (10), it is best not to focus exclusively on psychopathology when conducting research on seniors. Instead, studies should include more favourable characteristics of older adults, such as their potential for gaining higher self-esteem following successful completion of an educational intervention similar to the one tested herein.

Why implement a computer technology training intervention to improve the well-being of older adults? This could be a clever choice, given that, often due to fear of stigma, older adults are usually reluctant to seek traditional mental health services, even when in need (11), as stigma and other reasons such as lack of insurance and/or transportation present significant barriers to access. Consequently, interventions with an “apparent” exclusively educational focus, such as computer technology training, could serve the well-intended purpose of *bypassing* seniors’ common resistance to pursue mental health services. For computer illiterate older adults, receiving computer and internet training could have many benefits, as use of this technology in older age has been significantly linked to positive aging and seniors’ adaptation to the aging process (12). Positive uses of the internet in older age (and at all ages) include: communicating with loved ones, exploring hobbies/interests, accessing community resources, and increasing socialisation (e.g., meeting people through bulletin boards and chat rooms). There is an array of other benefits to using the internet in older age, as its utilization affords access to health literacy and ultimately to health action (i.e., taking control of one’s health at the community level). Indeed, using the internet is critical for gathering consumer health-related information on websites such as www.webmd.com (13), as well as for geriatric healthcare delivery and the prevention of age-related impairments (14). Overall, online activities can provide a means to access a variety of information as well as control interactional choices (15), which could contribute to increased well-being, as tested herein.

The first logical step when planning a computer training intervention, prior to testing its effects on well-being, is to test its impact on computer technology beliefs such as computer attitudes and self-efficacy.

In the present study, we also posed the question: “Is it possible to extend the benefits of our computer technology intervention to mental health *under a controlled condition*?” Indeed, if individuals, at any age, are exposed to a positive and successful training experience in which they feel competent and able to master a task, they may generalize such a successful experience to other areas. This, in turn, could positively impact global self-esteem and mental health (16, 17).

Concerning prior findings of interventions that addressed computer attitudes and computer self-efficacy, our laboratory obtained positive results, described in a 2008 article by the first author (18) on a pilot study with 32 seniors in which computer technology training significantly improved computer attitudes and computer self-efficacy. Such a theoretically- and empirically-based endeavor was conceptualized as a Phase I trial study that did *not* target mental health factors. Nonetheless, it was a critical step toward ascertaining whether our intervention could have beneficial effects on seniors; these findings were replicated by the first author and her colleagues in 2011 in a randomised controlled trial on a larger sample (19). These results are in line with findings from other laboratories that demonstrated the efficacy of computer training at enhancing: a) seniors’ computer attitudes (20-24); b) older adults’ level of comfort with computer use after attending a SeniorNet computer lab class (25); and c) young students’ computer self-efficacy (26, 27). Intervention studies on older adults’ computer self-efficacy are practically non-existent, with a few exceptions: 1) a recent study showing that computer training and internet use by cognitively intact older adults significantly increased their computer-related self-efficacy (28) and 2) the first author’s aforementioned two studies. Moreover, information on this outcome variable with regard to ethnically diverse senior populations is scarce. If training people in computer and internet use can improve their computer attitudes and computer self-efficacy, then, for the mental health-related reasons discussed above, older adults – and especially ethnic minority seniors (often neglected in research investigations) – have a clear need for this training.

A particular focus of the present intervention was the potential of one-on-one computer technology training to improve mood symptomatology. Regarding the theoretical foundation of this supposition, two related theories apply to this discussion: 1) a resource-related mental health theory by Hobfoll and Wells (29) and 2) a theory of conservation of resources in older age (30) by Gatz. Briefly, regarding the first theory, Hobfoll and Wells view resources available to seniors at both an individual level (e.g., personal health) and at a broader level (e.g., technological resources, internet) as having a significant impact on mental health outcomes. Similarly, the second theory by Gatz argues in favour of paying careful attention to the psychosocial resources of older adults to explain mental health disorders. In our case, learning computer/internet use could be added to seniors’ resources and consequently decrease their depressive symptomatology.

However, results of geriatric interventions targeting mood symptomatology as well as self-esteem through computer/internet training have produced conflicting results and failed to identify a well-validated intervention that could address these clinical issues. Some research findings suggest that computer/internet training administered to seniors yields a reduction in depressive symptomatology (31-33). However, some scholars did not identify significant depression-related improvements in their senior trainees (24), while others, following 9 hours of seniors group training over 2 weeks, only detected a trend that was not statistically significant at the .05 level (15). One reason for this lack of significance may be the group training format or the limited 2-week duration. Also, the results of a more recent study with a sample similar to ours in terms of health status and independent living status (34) showed no improvements in depressive symptomatology following two weeks during which three 4-hour training sessions were provided to the intervention group. Moreover, computer/internet training has the potential to enhance elders’ self-esteem (35), but again the scarce literature on this topic is divisive. For instance, while the findings of a study showed improved self-esteem among seniors who became computer users upon receiving weekly personal training over the course of 3 months (31), another investigation failed to demonstrate significant self-esteem changes among older adults who received a three-day one-on-one computer/internet training (24). We could speculate that maybe the short duration of the training, and/or seniors’ high level of psychological adjustment at baseline, affected the aforementioned results, but research is still needed in order to clarify this issue. It should be noted that the outcome of the aforementioned two studies conducted by the first author using computer technology training provided *no* indication of whether the intervention was capable of ameliorating seniors’ mental health, although the findings indicated significant post-training improvements in computer technology beliefs.

As to the present study’s hypotheses, research by the first author targeting the enhancement of computer attitudes and self-efficacy via the same computer technology training implemented in the current study (19) was successful. Likewise, we expected to find significant post-test improvements in trainees on both computer attitudes and self-efficacy at the end of the intervention. Moreover, computer technology attitudes and computer self-efficacy were hypothesized to be significantly related, based on prior findings on this topic (36) and given that these two variables are both dimensions of computer technology beliefs. We also chose a non-pathological variable - self-esteem - and hypothesized significant improvements in this factor in the experimental trainees by the end of our training. Specifically important in the field of ethnogeriatric psychiatry, we chose depressive symptomatology as an outcome variable that may be potentially amenable to positive changes through our training, expecting significant improvements only in experimental participants’ depressive symptomatology. We based this decision on the available literature in support of this hypothesis, and in particular on the encouraging results of an aforementioned study conducted in Israel by Shapira, Barak, and Gal (33) with Hebrew-speaking older adults (mean age=80.25 for the experimental group and 82.60 for the control group) using a very similar sample size (i.e., 22 experimental older adults and 26 controls) to that of our study. Shapira *et al*.’s findings showed significant improvement in depressive symptomatology following 15 weeks of computer training and internet use. When compared to our study, several elements were different, as their training was more than double the length of our intervention (while most other studies implemented an even shorter training than ours) and was presented in a group format versus a one-on-one structure. Also, the sample was ethnically homogeneous, as opposed to our ethnically diverse sample. Thus, testing the current intervention adds to the available empirical literature on one-on-one computer technology training and its effects on older ethnic minorities.

## METHOD

### Participants

We recruited 60 community-dwelling older adults, age 51 to 92 years (mean=69.12). They volunteered to be in the study (like the participants in the vast majority of research in this area) and were residents of Los Angeles County, California, United States of America (U.S.A). A variety of sampling strategies were used by the interviewers/research assistants (RAs), including purposive sampling (i.e., using their connections in their ethnic communities) and snowball sampling (i.e., mentioning to research participants that we were looking for referrals to other older adults who could participate in this research). Our goal was to overcome some of the limitations of prior research in this area by gathering an ethnically diverse sample and attempting to include isolated older individuals who knew at least one person in their community who could refer them to us. Adopting these sampling techniques allowed us to maximize the chances of obtaining a representative sample of older men and women residing in Los Angeles County, a very ethnically diverse area. We advertised this research project at several locations including stores, churches, senior centres, and senior apartment complexes. None of the respondents was recruited at a clinical facility or via a medical referral, the significance of which will be addressed later in this discussion.

The following inclusion criteria were established: 1) being at least 50 years-old; 2) being fluent in English if this was their second language (in order to avoid further confounding our findings with levels of acculturation); 3) being willing and able to attend all six sessions of our one-on-one training (even if our control/waitlist participants first received six weekly visits, not the training); 4) staying in the area for the next two months, and (5) being able to access a computer at their home. This last criterion was necessary in order for all our respondents to reap the benefits of our training after the study was completed (including the training of the waitlist participants), in accordance with the research ethics principle of offering training only for skills that can be used feasibly in the long run. Indeed, it has been documented that studying computer technology techniques is not beneficial to older adults unless they practice these skills on a regular basis (37), because computer use needs to be learned by way of both action and practice (38). Exclusion criteria were: 1) residing in an institutional setting; 2) being unable to grant informed consent (i.e., not being fluent in English enough to fully comprehend the content of the consent form); and 3) having more than ‘minor’ computer technology experience, i.e., having turned a computer on and off or having been exposed to other people utilizing computer technology to write documents or use the internet. As a result of implementing such criteria, all our participants were computer and internet illiterate (i.e., non-users of the technology taught in our training).

### Measures

Our four outcome variables were assessed twice (pre- and post-test) while the rest of the variables were assessed only at baseline, to avoid burdening older adults. To quantify the socio-demographic and computer-related issues required for inclusion/exclusion purposes, we used a list of items that covered all the inclusion and exclusion criteria; this is the same list as the one used in the first author’s 2011 study (19). It contains variables of interest including age, education, household income, and ethnic background, as well as computer ownership, access to a computer, prior computer experience, and ability to e-mail.

To calculate computer technology attitudes, we utilized the latest version of the *Older Adults’ Computer Technology Attitudes Scale *[*OACTAS* (18, 19)], which has achieved strong reliability results, as reported in the aforementioned 2011 study (Cronbach’s α reliability=0.92). All its 17 items are negatively worded, in an attempt to elicit candid responses to computer technology questions from computer illiterate individuals. Responses are coded on a 7-point Likert-type scale from ‘-3’ ‘strongly disagree’ to ‘+3’ ‘strongly agree’; the scores are reversed before conducting data analyses, in order to have higher numbers denote more positive computer technology attitudes.

The 30-item *Computer User Self-Efficacy Scale* (39) was used to assess computer-self-efficacy; according to its authors, the Cronbach’s *α* of this measure is 0.97 and the test-retest reliability coefficient is 0.86. Items are rated on a 6-point Likert-type scale ranging from ‘1=strongly disagree’ to ‘6=strongly agree’. The only minimal adaptation of this tool involved deleting an irrelevant introductory item applicable only to college students.

To quantify health status (which was done for descriptive purposes only), we used a very short version of a well-validated health measure, the *12-item SF-12 Health Survey*, which is a sound measure of health status (40). Its multi-item scale assesses 8 health concepts: physical functioning, role limitations due to physical health problems, bodily pain, general health, vitality (energy/fatigue), social functioning, role limitations due to emotional problems, and mental health (psychological distress and psychological well-being). This measure has high test-retest reliability scores (0.76-0.89). To minimize burdening our older research participants, given that physical health was not one of our outcome variables, we asked only the first two questions: 1) self-rated health and 2) ease of engaging in moderate activities of daily living, including moving a table, pushing a vacuum cleaner, bowling, or playing golf.

Self-esteem was measured using the *Rosenberg Self-Esteem Scale:* a 10-item, 6-point Likert-type scale measuring basic feelings of self-worth (41, 42). Its Cronbach’s α internal consistency is 0.74 among non-institutionalized seniors (43). It has been previously utilized with older adults (42) and recognized in the literature as an appropriate scale for measuring global self-esteem in older age (44). Its utilization allowed the operationalisation of our respondents’ global self-esteem at baseline and follow-up assessments.

We used the *Beck Depression Inventory – II (BDI-II)* to assess depressive symptomatology; it contains 21 sets of 4 statements that describe varying intensities of somatic and cognitive-affective symptoms of depression (45). Respondents choose the one statement from each group that best describes how they have been feeling for the past 2 weeks. This tool is appropriate for use with geriatric samples (46) and was utilized to operationalise changes in mood symptomatology before and after our intervention. Based on findings of a study on a depressed geriatric sample (47), the BDI-II’s internal consistency is very high (α=0.90), and gender, ethnicity, or age are not significantly related to the total scores on this measure. This is ideal for our sample, as it is comprised primarily by women. An appropriate BDI-II’s cut-off score for significant depression among geriatric populations is 10, as using this score in a study on cognitively intact older adults led to 96.30% sensitivity in correctly identifying depressed and non-depressed subjects (48).

### Research design, procedures, and computer technology training

In the current pilot Phase II Efficacy study, we tested, for the first time, the potential positive impact of our one-on-one, manualized training intervention on depressive symptomatology and self-esteem in older age. We also tested changes in computer attitudes and computer self-efficacy, to verify whether these changes were necessary before improvement in well-being could occur. We conceptualized these four variables into two themes, i.e., theme 1) computer attitudes and computer self-efficacy, and theme 2) self-esteem and depressive symptomatology. As done in the first author’s two prior studies, the present research was conducted following the recommendations of the first author’s original research model relative to the implementation of high-quality research on community-dwelling older adults (49). This model pays particular attention to avoiding methodological challenges common to this type of geriatric research.

We conducted a randomised, controlled 6-week intervention; our research procedures were in accordance with the ethical standards of the Institutional Review Board of California State University Northridge concerning research employing human subjects. The one-on-one computer training programme imparted in this project was designed by the first author (18) to enhance older adults’ computer technology attitudes and self-efficacy. This is the first time that our laboratory has used this manualized one-on-one training for well-being/mental health enhancement purposes. Each older adult recruited for this study signed our consent form. Every respondent was assigned an RA to perform the pre- and post-tests as well as to train him/her; at baseline, RAs collected data on socio-demographic attributes, physical health, and on the four outcome variables. After six weeks, all participants were re-tested on the four outcome variables, including the control subjects, who were trained following completion of the second assessment. Control participants were visited by their RA for one and a half hours per week without engaging in training, in order to match the amount of attention given to participants in both groups. At post-test, our experimental subjects were asked to e-mail their RA, in the presence of the trainer but without any assistance (to make sure that the trainee had indeed sent the email). All experimental subjects were able to complete this email task.

The first author trained all RAs to ensure their effectiveness as one-on-one computer trainers; the latter were asked to avoid deviating from training manual instructions and, for the purpose of quality assessment, to keep a diary of the training experience with each trainee and to document anomalies or deviations from the instructions. No substantial deviations were reported, as evidenced by inspections of the diaries’ content by the second author and several RAs. The training protocol was implemented on a desktop computer, at locations identified by the participants as being convenient, including the Department of Psychology at California State University Northridge and several libraries in the area. The first author wrote the training manual in order to standardise the training procedure. Its content has been described in detail elsewhere in the literature (18-19). Generally speaking, in our training, we aimed at maximizing trainees’ active participation in learning computer and internet use and asked RAs to provide fast feedback to trainees on their progress during training. After using the same training manual, the RAs in the aforementioned 2011 study by the first author reported that they (as well as their trainees) found the manual and the related training easy to follow and comprehend. Possibly due to all these procedures being in place, we did not experience any subject loss from pre- to post-test.

### Power Analyses

A-priori power analyses were run with G*Power Version 3.1.5 (50) in order to identify the number of participants needed for each group, based on the effect sizes (*ES*; η^2^ converted to Cohen’s ƒ) reported in several randomised control trials of internet training interventions assessing similar outcome variables to those of the current study. According to Cohen’s (51) recommendations for adequate power (i.e., >0.80), for theme one, the experimental and control groups each required 12 participants for the computer self-efficacy variable and 6 participants for the computer attitudes variable, according to estimates provided by Laganá (18) [*ES_computer_*_self-efficacy_ = 0.94; *ES_computer attitudes_* = 2.06]. As for theme two, the experimental and control group each required 13 participants for the self-esteem variable according to Billipp’s findings (31) [*ES_self-esteem_*=0.87] and 29 participant for depressive symptoms according to estimates provided by Shapira, Barak, and Gal (33) [*ES_depression_*=0.55]. Thus, we chose the upper limit of 30 participants per group to ensure our ability to detect these effects. Our a-priori power analyses suggest that the effects for these interventions are subtle.

### Analytic strategy

In line with the analytic strategy adopted in the 2011 randomised controlled study on only computer attitudes and computer self-efficacy, we intended to run two separate MANCOVAs, one per theme not correlated over 0.30, using the Statistical Package for the Social Sciences. The first MANCOVA would allow the testing of post-training changes in the two computer-focused outcome variables, the second one in the two mental well-being variables. However, if any of these two sets of two variables had been correlated over 0.30, we intended to conduct Roy-Bargmann’s stepdown analyses instead (involving separate ANCOVAs), thus complying with the methodological recommendations of well-respected statistical sources (52). To avoid losing power in our analyses, given the limited size of the sample, we did not plan to use any other covariates than those dictated by the data analyses procedures, i.e., pre-test scores when predicting post-test scores in addition to another covariate if required by Roy-Bargmann’s procedures (in the result section below, we have detailed this occurrence).

## RESULTS

Concerning the frequency findings, Table 1 illustrates the demographic and health characteristics of the sample. We recruited 42 women and 18 men; only about 1/3 of our sample self-identified as White. As to perceived physical health status, 31.7 % of the sample self-rated physical health as fair, 48.3% as good, 18.3% as very good, and 1.7% as excellent. The intercorrelation matrix, which contains Pearson product-moment correlation coefficients, is reported in Table 2. It should be noted that we did not use the variable income in our analyses, as over one fourth of the sample either did not know the answer or refused to answer this question. Instead, we used the variable education as its proxy, given that education and income are often used as proxies for socioeconomic status (53).

**Table 1:** Characteristics of the sample

Variable	Mean (Standard Deviation)	%

Age	69.12 (10.37)	
Ethnicity		
European-American		32.7
Mexican-American		20
Other Hispanic/Latino		7
Asian-American		15
Middle Eastern		22
American Indian/Native American		3.3
Education		
Less than High School		33.3
Graduated from High School		35
Completed Trade School		6.7
Some college		13.3
Bachelor’s degree		5
Some graduate school		1.7
Master’s degree		1.7
Ph.D., M.D., and/or J.D.		1.7
Refused to Answer		1.7
Yearly Income		
Less than $20,000		20
$20,000-$39,000		28.3
Over $40,000		25
Refused to Answer		26.3
Self-rated general health	3.10 (2.13)	
Impairment in activity of daily living	2.13 (0.68)	
Baseline computer attitudes	71.09 (23.09)	
Baseline computer self-efficacy	81.60 (26.53)	
Baseline self-esteem	16.10 (3.80)	
Baseline depression	10.00 (7.24)	

**Table 2:** Zero-order correlations between demographic, independent, and dependent variables

Variable	Age	Sex	Education	Self-rated general health	Impairment in activity of daily living	Baseline computer attitudes	Post-test computer attitudes	Baseline computer self-efficacy	Post-test computer self-efficacy	Baseline self-esteem	Post-test self-esteem	Baseline depression	Post-test depression

Age	-	0.07	-0.11	-0.07	-0.37^**^	-0.02	0.04	-0.02	-0.13	-0.21	-0.06	0.01	-0.01
Sex		-	0.31^*^	0.16	0.14	-0.03	0.06	0.05	0.06	0.08	0.15	0.12	0.06
Education			-	0.07	0.20	0.01	-0.06	0.21	0.28^*^	0.01	0.16	0.15	0.03
Self-rated general health				-	-0.36^**^	0.13	0.10	-0.16	-0.05	0.33^**^	0.40^**^	0.43^**^	0.36^**^
Impairment in activity of daily living					-	-00.30^*^	-0.15	-0.16	-0.05	0.04	-0.27^*^	-0.26^*^	-0.30^*^
Baseline computer attitudes						-	0.69^**^	-0.56^**^	-0.40^**^	0.01	0.27^*^	0.18	0.25
Post-test computer attitudes							-	-0.51^**^	-0.56^**^	0.01	0.18	0.17	0.23
Baseline computer self-efficacy								-	0.73^**^	-0.05	-0.13	-0.13	-0.23
Post-test computer self-efficacy									-	0.01	0.13	-0.14	-0.30^*^
Baseline self-esteem										-	0.58^**^	0.08	0.04
Post-test self-esteem											-	0.38^**^	0.31^*^
Baseline depression												-	0.85^**^
Post-test depression													-

* p<0.05;

** p<0.01

The means of the four outcome variables are displayed by group in Table 3. Baseline and post-intervention mean scores on self-esteem and depression were similar in the two groups. Based on the typical cut-off score used in the geriatric depression literature (48), the control group fully qualified at baseline as being significantly depressed, given that the mean depression scores were above the clinical cut-off score of 10. The experimental group exhibited depression scores that were almost clinically significant, as the mean score was over 9. However, regarding the computer-related outcomes (i.e., computer self-efficacy and computer attitudes), the two groups’ baseline means appeared markedly different. To test whether these differences were statistically significant, we compared group means by conducting two separate t-tests. The findings suggested no significant differences between the experimental and control participants at baseline for computer attitudes [*t*(58)=1.06, *p*=0.29] or computer self-efficacy [*t*(58)=-1.86, *p*=0.07], which suggests that both groups held statistically similar beliefs and attitudes regarding computer technology at baseline. Given these results, it was methodologically adequate to compare our two groups on all four outcome variables.

**Table 3:** Pre- and post-intervention means of the four dependent variables by group/condition

Variable	Control group baseline mean	Control group post-intervention mean	Experimental baseline mean	Experimental post-intervention mean

Computer attitudes	74.25	73.79	67.93	68.65
Computer self-efficacy	75.37	75.95	87.83	108.18
Self-esteem	15.76	16.46	16.44	15.66
Depression	10.96	11.40	9.04	6.88

As previously mentioned, we planned on conducting either two MANCOVAs with two outcome variables each - one for the computer-related theme and one for the mental well-being theme - or a series of four separate ANCOVAs if, applying Roy-Bargmann’s suggestions (52), these two sets of variables had correlations over 0.30. Relative to our computer-related theme, we found a high post-test computer attitudes and computer self-efficacy correlation (*r*=-0.56, *p*<0.001), which required the use of two separate ANCOVAs.

In the first analysis/step of the Roy-Bargmann’s procedure for the computer-related theme, we implemented an ANCOVA, controlling for baseline attitudes and baseline computer self-efficacy. This was done in order to test for the presence of training improvements for computer attitudes in the experimental participants, as this variable was theoretically the first computer-related factor to consider as potentially being impacted by our intervention. Homogeneity of variance and regression assumptions were not met, and there were no group differences with regard to computer attitudes [*F*(1, 56)=0.01, *p*=0.93, *η^2^*<0.01].

**Figure 1:**
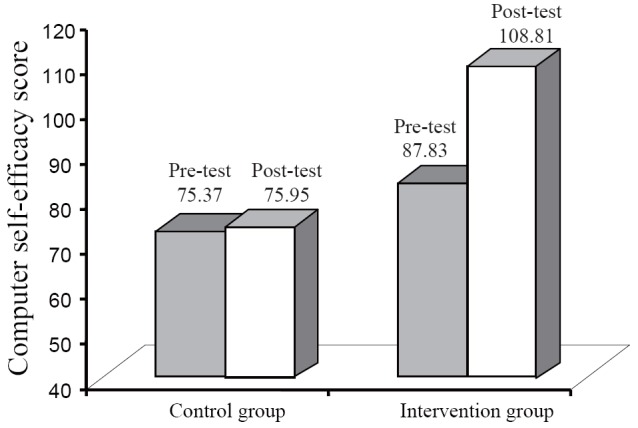
Visual representation of the self-efficacy results.

Concerning the second step of this procedure, to test post-test group differences in computer self-efficacy due to training, we controlled for baseline computer attitudes and baseline computer self-efficacy as well as for post-test computer attitudes, in line with the Roy-Bargmann’s procedure. Both the homogeneity of variance and the test of the homogeneity of regression assumption were met. The results of the ANCOVA showed a significant main effect for group at follow-up (as illustrated in Figure 1), with the experimental group reporting higher computer self-efficacy compared to the control group [*F*(1,56)=22.98, *p*=0.001, *η^2^*=0.01].

The mental health/well-being theme of the study was tested via the final two ANCOVAs, given that the correlation between depression and self-esteem was over 0.30 (i.e., *r*=0.31, *p*<0.05). In step 1 of this procedure, the outcome variable to test for training-related improvements was self-esteem, which we conceptualized as the first well-being factor to possibly be impacted by our intervention. Thus, in this Roy-Bargmann step-down ANCOVA, we targeted post-test self-esteem while controlling for baseline self-esteem and baseline depression. Like in the first ANCOVA, the homogeneity assumptions were not met, and we did not detect any significant differences concerning self-esteem between the experimental and control groups [*F*(1, 56)=11.07, *p*=0.26, *η^2^*<0.01].

**Figure 2:**
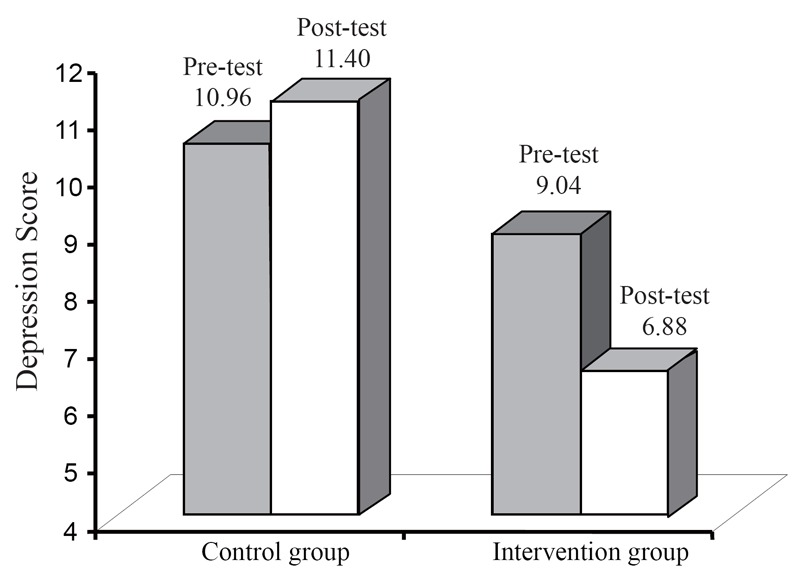
Visual representation of the depressive symptomatology results.

In the final step, we tested post-test group differences in depressive symptomatology while controlling for baseline depressive symptomatology and baseline self-esteem as well as for post-test self-esteem. The homogeneity of variance and the test of the homogeneity of regression assumption were met. Results demonstrated a significant main effect for group at follow-up (as depicted in Figure 2), with the experimental group reporting significantly lower depressive symptomatology compared to the control group [*F*(1, 55)=9.06, *p*=0.004, *η^2^*=0.02].

We also conducted pre-post reliability analyses for the four outcome variables, in order to rule out measurement error as a possible confound for our result, as poor reliability introduces error and may decrease the ability to detect an effect. Our measures were robust, given the modest sample size, considering that a result of 0.80 and above indicates adequate reliability (51). For computer attitudes, the pre-test internal consistency coefficient showed excellent results at 0.90 and at post-test was almost identical at 0.89. Regarding computer self-efficacy, the pre-test internal consistency coefficient was excellent at 0.92 and at post-test appeared somewhat improved at 0.95. For self-esteem, the pre-test internal consistency coefficient was 0.79 and at post-test was 0.81, indicating adequate reliability. For BDI/ depressive symptomatology scores, the pre-test internal consistency coefficient was .89, and at post-test was 0.91; these results are indicative of robust clinical utility.

## DISCUSSION

In this study, we compared two groups of community-dwelling seniors to investigate the effects of the first author’s manualized, one-on-one computer technology training for older adults on two themes, i.e., theme 1) computer attitudes and often-related self-efficacy and theme 2) self-esteem and its well-known correlate, depressive symptomatology. Alarmingly, the mean score on depression for this sample was at the clinical cut-off score of 10; thus, our intervention was indeed needed. As a result, the present investigation became a randomised controlled study for a *clinical* sample. Although the two variables in each theme were significantly related, only one of them per theme showed to be amenable to positive changes in older age as a function of computer and internet training. Our findings have psychiatric relevance given that, in addition to positively impacting computer self-efficacy, our computer technology training had beneficial effects on depression in the experimental group, pushing this group’s mean depression score well below the clinical cut-off score. This is noteworthy, considering the relatively small sample size of our study.

The findings of the first ANCOVA were somewhat perplexing, as they contradict the results of the aforementioned studies by the first author and those of other researchers (20-24). The results of this ANCOVA also oppose prior empirical evidence (55) suggesting that negative computer attitudes in older age can stem from having limited computer technology experience, as our experimental participants were given several weeks of experience with this technology. More research is needed to replicate this finding; perhaps it is indeed possible for older adults *not* to improve their views of computer technology in older age and *still* reap the benefits of this technology.

The results of the ANCOVA on computer self-efficacy were in line with our lab’s prior findings (18, 19) as well as with results from other laboratories (28). The significantly improved computer self-efficacy among experimental participants may be attributable to improvements in sense of mastery of the tasks learned during training, such as how to surf the internet and send emails to loved ones. If liking this technology did not improve as a result of our intervention, *self-assurance regarding computer usage* certainly did. Considering the already relatively high baseline mean of computer self-efficacy for experimental participants, the present findings suggest that, if we provide computer and internet training to computer-illiterate, ethnically diverse older adults, they can *still* significantly increase their computer self-efficacy as a function of training *regardless* of their level of pre-training confidence about being able to learn and use this technology. As part of our inquiry regarding a possible significant relationship between computer self-efficacy and depression, we asked this question of the experimental group only, as this is the group in which we detected significant changes on these two variables as a result of our training. However, upon conducting ancillary analyses to examine the relationship of computer self-efficacy to depression both at baseline and after the training, we did not obtain significant relationships at pre-test(*r*=-0.16, *p*=0.39) nor at post-test (*r*=-0.12, *p*=0.54). Furthermore, post-hoc partialcorrelational analyses, controlling for the influence of baseline computer self-efficacy and baseline depressive symptomatology, further revealed that post-test computer self-efficacy among trainees was not significantly related to post-test depression scores (*r*=-0.15, *p*=0.46). This result supports prior findings on depression and internet self-efficacy not being significantly related among undergraduate students (56) and extends them to an ethnically diverse geriatric population.

The mental health/well-being theme of our study included two variables, self-esteem and depression. Self-esteem failed to show significance, in line with prior literature showing that this variable does not improve with computer training in older age (24) but in contrast with research findings by Billipp (31) showing that it becomes significantly higher in older age as a result of receiving weekly personal training over the course of 3 months and becoming regular user. Perhaps Billipp’s aforementioned positive results were due to the fact that trainees became computer users for several months, while our trainees were not monitored in their computer use aside from participating in our intervention. Future research is needed to clarify this point.

Depressions scores that, prior to training, were on the cusp of clinical significance in our experimental group, were very positively impacted by our training; this is the most clinically relevant outcome of the present study. Indeed, experimental participants started the training at levels of depression that were statistically comparable to those of the control subjects (who were significantly clinically depressed). Yet, as a result of training, trainees’ depression scores were significantly reduced. It is clinically noteworthy that the percentage of significantly depressed experimental subjects was reduced by 20%, i.e., from 36.7% at baseline to only 16.7% after the intervention. Our depression result supports some prior findings with sample sizes similar to ours [e.g., 22 experimental older adults and 26 controls (33)] but conflicts with results reported by other researchers (34), which showed no significant depression reduction as a function of computer training in older age. Furthermore, the total sample’s mean score of 10 on depression at baseline suggests that many non-institutionalized, ethnically diverse older adults are living with untreated depression: based on this number, depression should become a very high-priority target of community health programs for older adults of all ethnic backgrounds. More research is needed to corroborate our depression findings.

### Limitations of the study

Several limitations of this study must be acknowledged, such as the previously noted modest size of its sample - although some of the aforementioned geriatric studies gathered comparably sized samples or even smaller samples. Furthermore, although most of our participants were non-White, they all resided in urban or suburban areas of Los Angeles County, which limits generalization of our results to seniors residing in rural areas or those living outside of the United States. Additionally, the relatively brief 6-week intervention may not have allowed us to truly capture the effect of internet training on our two themes over time (i.e., longitudinally). Thus, future research directions may include examining internet training and its effect on computer attitudes and related self-efficacy as well as global self-esteem and depressive symptoms in order to 1) quantify the effect size over time and 2) identify the dose-response of this intervention. Also, the fact that men comprised only 30% of our sample precluded the possibility of conducting statistically meaningful gender comparisons. In future studies, interested researchers should investigate whether gender plays a significant role in the effects of similar interventions, as the available evidence in this area points to older women being under-represented online and not reporting reaping substantial benefits from using the internet (57). In order to substantiate the present results and related explanations, there is certainly a need for more adequately powered future investigations that should ideally include the assessment of anti-depressant medication use and the utilization of other treatment modalities for depression. Concerning the strength of our results, in the present study, we reported effect sizes as eta squared, as opposed to partial eta squared; there are several advantages to using eta squared, as described in the literature (58). Our effects for both computer self-efficacy and depression were *not* statistically trivial, according to Cohen (51). However, as evidenced by the findings of our power analyses, the effects for these types of interventions tend to be subtle, which may also reflect the typical use of small samples in this area of research.

Briefly, regarding cultural considerations, to our knowledge, this is the first U.S.A.-based geriatric study on the effects of computer technology training in which about 68% of the subjects recruited are non-White. Given that Hispanics represented 27% of our sample and Asians 15%, for a total of 42% (Whites were 32.7%), cultural issues should be considered in the discussion of our depression findings. To cite just one cultural value, due to space limitations, *familismo* refers to the importance of close family relations and intergenerational exchanges of social support. It is a particularly strong value among Hispanic and Asian older adults (59). In line with the concept of familismo (not assessed here as it was beyond the scope of this study), increased use of the internet may have created an important avenue for increased social support and networking in older age, allowing older Hispanic and Asian participants, for instance, to establish and maintain contact (over the course of the 6 weeks of training) with grandchildren who moved away to attend college or other family members not living nearby. This new opportunity for strengthening family contacts via online interaction (not assessed herein as it was not within the scope of this research) might have positively affected their mental health and well-being, and could have been a factor impacting our depression findings. Research in this area is needed to experimentally test this conjecture. Also, in future studies, the recruitment of ethnically diverse older adults presenting with a range of psychopathology of different kinds would be ideal, as different mental health pathologies could be tested for mitigation post-training. This would allow researchers to test factors potentially affecting improved psychiatric symptomatology, such as increased online contact with loved ones, engagement in uplifting online activities, or enhanced community interaction as a result of acquiring community-related information or personal contacts online.

## CONCLUSIONS

From a clinical standpoint, this investigation was a controlled study on a clinical sample. Although we did not intend to recruit a clinical sample, on average, our 60 community-dwelling seniors had significant depression at baseline, which was markedly ameliorated to the point of no longer being clinically significant for 20% of the experimental group. Our findings suggest that computer and internet training can lead to higher levels of computer self-efficacy and improved mood symptomatology among older, computer-illiterate seniors from a variety of ethnic backgrounds. Gains on these two outcome variables were not reported by our control subjects. If our intervention is further confirmed as having psychiatrically significant impact, the relatively inexpensive form of computer technology training used in this study could become an effective yet *neutral/non-pathologizing intervention* for the reduction of mood psychopathology (ideally in conjunction with needed psychiatric or other pertinent treatment). Such an intervention would be consistent with the aforementioned values related to avoiding the stigma of receiving mental healthcare often held by ethnically diverse senior populations.
